# High-resolution iris and retinal imaging in multisystemic smooth muscle dysfunction syndrome due to a novel Asn117Lys substitution in ACTA2: a case report

**DOI:** 10.1186/s12886-020-01344-w

**Published:** 2020-02-24

**Authors:** Aisling B. Mc Glacken-Byrne, David Prentice, Danial Roshandel, Michael R. Brown, Philip Tuch, Kyle S.-Y. Yau, Padma Sivadorai, Mark R. Davis, Nigel G. Laing, Fred K. Chen

**Affiliations:** 1 Centre for Ophthalmology and Visual Science (incorporating Lions Eye Institute), The University of Western Australia, 2 Verdun Street, Nedlands, WA 6009 Australia; 2 Department of General Medicine, Royal Perth Hospital, Perth, Western Australia Australia; 3 Perron Institute, Nedlands, Western Australia Australia; 4 Hollywood Medical Centre, Nedlands, Western Australia Australia; 5 Harry Perkins Institute of Medical Research, QEII Medical Centre, Nedlands, Western Australia Australia; 6 Department of Diagnostic Genomics, PathWest Laboratory Medicine, QEII Medical Centre, Nedlands, Western Australia Australia; 7 Centre for Medical Research, The University of Western Australia, Crawley, Western Australia Australia; 8 Department of Ophthalmology, Royal Perth Hospital, Perth, Western Australia Australia; 9 Department of Ophthalmology, Perth Children’s Hospital, Nedlands, Western Australia Australia

**Keywords:** Congenital mydriasis, Retinal artery tortuosity, OCTA, Adaptive optics, MSMDS

## Abstract

**Background:**

Congenital mydriasis and retinal arteriolar tortuosity are associated with the life-threatening multisystemic smooth muscle dysfunction syndrome (MSMDS) due to mutations in the gene, *ACTA2*, which encodes alpha-smooth muscle actin (α-SMA). Previous reports attributed MSMDS-related congenital mydriasis to the absence of iris sphincter muscle. Similarly, it has been hypothesized that abnormal proliferation of the vascular smooth muscle cells causes the marked tortuosity of retinal arterioles in MSMDS. In this report, high-resolution ocular imaging reveals unexpected findings that reject previous hypotheses.

**Case presentation:**

The proband is a 37-year-old female with a history of neonatal patent ductus arteriosus (PDA) ligation, left-sided choreiform movements at the age of 11 and a transient aphasia with right-sided weakness at the age of 30. Her older sister also had PDA ligation and congenital mydriasis but no neurological deficit up to age 41. Magnetic resonance angiogram demonstrated cerebrovascular lesions resembling but distinct from Moyamoya disease, characterised by internal carotid artery dilatation, terminal segment stenosis and absent basal collaterals. Their mother had poorly reactive pupils with asymptomatic cerebral arteriopathy resembling her daughters. All three had prominent retinal arteriolar tortuosity. The daughters were heterozygous and the mother was a somatic mosaic for a novel c.351C > G (p.Asn117Lys) transversion in *ACTA2*. Iris optical coherence tomography (OCT) showed a hyporeflective band anterior to the pigment epithelium indicating the presence of dysfunctional sphincter muscle. Adaptive optics retinal imaging showed no thickening of the arteriolar vessel wall whilst OCT angiography showed extreme corkscrew course of arterioles suggesting vessel elongation.

**Conclusions:**

In addition to the known association between Met46, Arg179 and Arg258 substitutions and *ACTA2*-related arteriopathy, this case illustrates the possibility that Asn117 also plays an important role in α-SMA function within the cerebrovascular smooth muscle cell. MSMDS-related congenital mydriasis is due to reduced iris sphincter contractility rather than its absence. Retinal arteriolar tortuosity might be due to longitudinal proliferation of arteriolar smooth muscle cells. The described cerebrovascular and ocular signs are consistent with predicted effects of the novel Asn117Lys substitution in ACTA2.

## Background

Iris abnormalities have long been recognised as part of a syndrome with a diverse life-threatening systemic manifestations including familial thoracic aortic aneurysm and dissection (TAAD) [1, 2], patent ductus arteriosus (PDA) [3, 4], aortopulmonary window (APW) [5], combined TAAD, PDA and brachytelephalangy [6], combined PDA and congenital cystic lung disease [7], moyamoya-like cerebral angiopathy with dolichoectasia of the internal carotid artery and PDA [8] and megacystis-microcolon-intestinal hypoperistalsis syndrome [9, 10]. The possibility of a genetic basis for these observations was confirmed by the report of an association between various heterozygous missense mutations in the gene encoding the protein alpha-smooth muscle actin (α-SMA), *ACTA2*, and inherited TAAD [11]. Subsequently, Milewicz et al. and others described a highly penetrant phenotype of a Multisystemic Smooth Muscle Dysfunction Syndrome (MSMDS, OMIM#613834) associated, specifically, with the Arg179 substitutions in α-SMA; characterised by congenital mydriasis, retinal artery tortuosity, livedo reticularis, ascending TAAD, coronary artery disease, PDA, APW, cerebral arteriopathy resembling but distinct from the classic moyamoya disease (MMD), periventricular white matter lesions, hypotonic bladder, malrotation and hypoperistalsis of the intestinal tract, and pulmonary hypertension [12–14].

It has been speculated that mutations at critical residuals on α-SMA results in a reduced rate of non-covalent polymerization of G-actin into F-actin, a double stranded filament essential for smooth muscle cell (SMC) contractility and rigidity [15]. Through either a primary or secondary defect in extracellular matrix production, the inefficient submembranous actin polymerization leads to proliferation of SMC in arterial walls and secondary luminal obstruction [15]. However, the mechanism by which *ACTA2* mutation leads to ocular SMC abnormality remains unknown. Previous studies hypothesized that the congenital absence of iris sphincter and dilator muscles may explain the observed permanent mydriasis unresponsiveness to pharmacologic dilating and miotic agents [12, 16–19]. The age-dependent retinal arteriolar (but not venular) tortuosity and aneurysmal-like corkscrew course was thought to be due to abnormal vascular SMC proliferation in arteriolar walls but this has not been examined in detail [17–19]. Herein, we use optical coherence tomography (OCT), OCT angiography (OCTA) and adaptive optics (AO) imaging to examine the iris and retinal phenotype of a family with a heterozygous Asn117Lys substitution in α-SMA to understand the structural basis of ocular manifestation.

## Case presentation

**Fig. 1:**
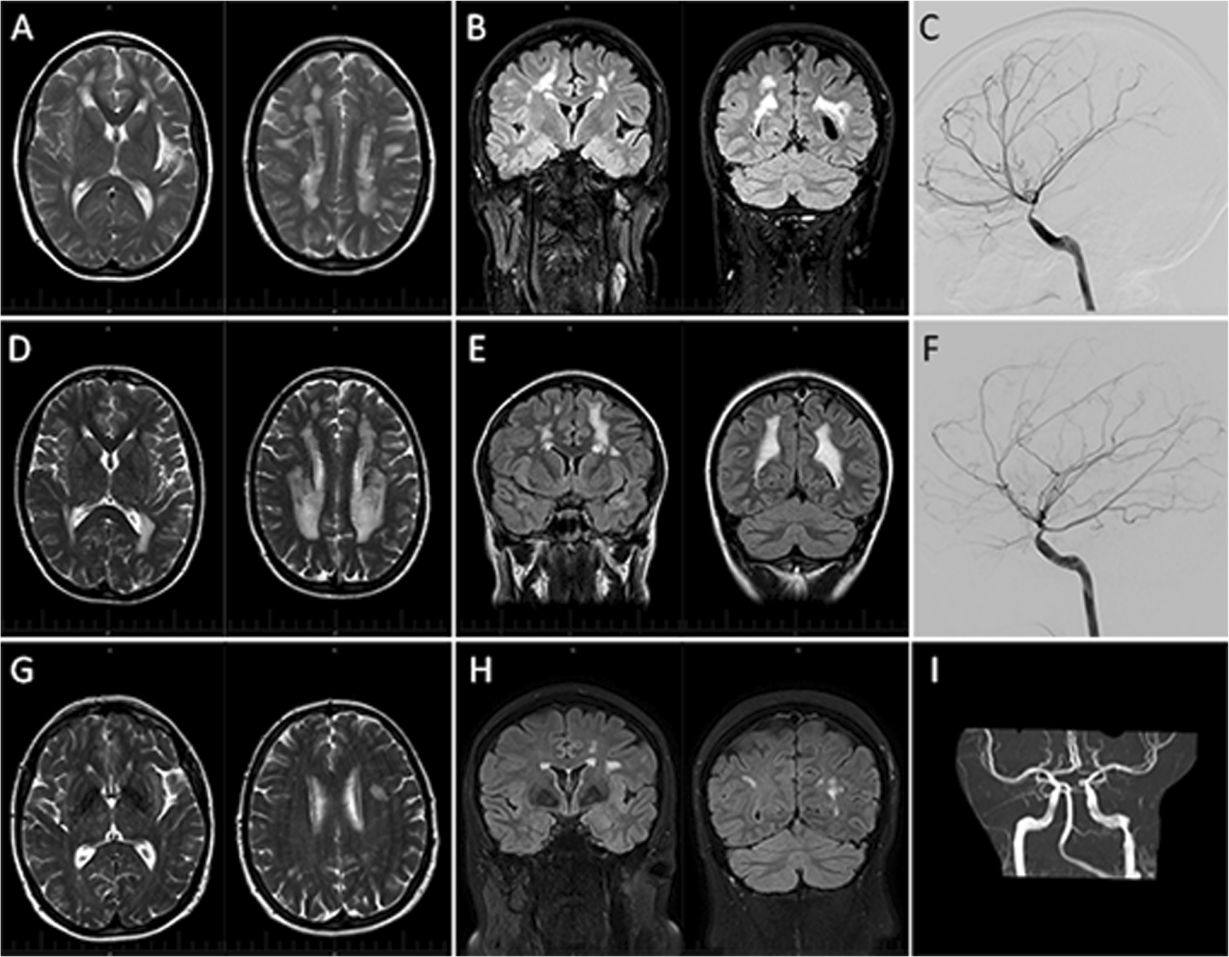
Proband’s axial magnetic resonance imaging at age 34 years showed extensive T2 hyperintensity in the periventricular and deep white matter of both cerebral hemispheres distributed predominantly in the deep water-shed regions whilst sparing the superficial water-shed territories (**a**). There was no sulcal FLAIR hyperintensity on coronal imaging (**b**). Catheter digital subtraction angiography (DSA) showed severe supraclinoid internal carotid stenosis, patulous petrous segment of the internal carotid artery, straightening of cerebral vessels and absent basal moyamoya collaterals (**c**). The proband’s older sister had similar distribution of T2 hyperintensity (**d**) and absence of sulcal FLAIR hyperintensity (**e**) at the age of 37 years. Her DSA showed similar a cerebral vascular anomaly that is distinct from moyamoya disease (**f**). The proband’s mother, aged 62 years, also had multiple foci of T2 hyperintensity in the deep and subcortical white matter of both hemispheres with physiologic calcification in the globus pallidi bilaterally (**g**). There was no sulcal FLAIR hyperintensity (**h**) and MR angiography showed fusiform dilatation of the internal carotid arteries within the proximal carotid canal bilaterally (**i**)

The proband is a 37-year-old white female a history of PDA ligation as a 6-week-old. A cerebral magnetic resonance imaging (MRI) scan performed at the time of acute onset of left-sided weakness and choreiform movements of the left arm at age of 11 revealed extensive periventricular deep white matter hyperintensity and a lesion in the right basal ganglia. Magnetic resonance angiography (MRA) a year later showed MMD of the anterior circulation. At age 26, a referral to the neurologist for further assessment of ongoing minor choreiform movements and left sided paraesthesia led to the observation of fixed-dilated pupils. At age 30, she represented with expressive aphasia and right-side weakness which resolved within 6 h. MRI/MRA demonstrated acute left middle cerebral artery (MCA) branch infarct involving left insula, inferior frontal gyrus and superior temporal gyrus. Subsequent cerebral angiography confirmed an occlusive cerebral angiography that is distinct from MMD characterised by severe tapering stenosis of the supraclinoid segment of the internal carotid arteries (ICA) with dilatation of their proximal portions, straightening of cerebral vessels and absence of basal collaterals (Fig. 1). Echocardiogram of the heart and aortic root and Doppler ultrasound of the lower limb arteries were normal and autoimmune serology was negative. Consideration was given to an external carotid-internal carotid bypass but this was not undertaken as she was asymptomatic. Therapy with aspirin was continued.

The older sister of the proband is a 41-year-old mother of 2 with a history of PDA ligation as a newborn. Fixed dilated pupils were noted as a child and diagnosed as congenital mydriasis by an ophthalmologist. At age 36, she presented to the neurologist with tension headache, a cranial computed tomography (CT) scan showing hypodensity white matter lesions. She had a normal neurological and systemic examination except for the non-reactive mid-dilated pupils. Her 2 children had reactive pupils. The proband’s mother, at the age of 66 years, has a history of hypercholesterolemia adequately controlled with statins. She was well and physical examination was essentially normal except for poorly reactive small pupils which dilate from 3 to 5 mm with 1% tropicamide. MRI/MRA performed in the older sister and the mother showed similar ischaemic and vascular changes to those in the proband (Fig. 1). Echocardiogram in all 3 patients revealed normal aortic root size.

Ophthalmic clinical findings are summarised in Table 1. Ophthalmic investigation included iris and retinal spectral-domain OCT using the Spectralis HRA + OCT (Heidelberg Engineering, Heidelberg, Germany). Ultra-widefield fundus photography was performed using the California (Optos plc, Dunfermline, Scotland). Optical coherence tomography angiography (OCTA) was performed using the AngioVue Avanti XR (Optovue Inc., Fremont, USA) in AngioRetina mode. ReVue software (Version 2017.1.0.155) was used for reducing motion artefacts and en face view reconstruction.

**Table 1:** Clinical features of the three affected individuals

	Proband	Older sister	Mother
Pedigree	III:1	III:3	II:5
Current age (years)	37	41	62
History of PDA repair	Yes	Yes	No
History of stroke (age in years)	Twice (11 and 30)	None	None
Echocardiogram (performed at age in years)	31	36	64
Neuroimaging (performed at age in years)	MRI/MRA (12, 26, 28, 30, 34, 37) cerebral angiography (30)	MRI/MRA (36, 37) cerebral angiography (36)	MRI/MRA (62)
Visual acuity (Snellen) OD; OS	6/6; 6/6	6/6; 6/6	6/6; 6/6
Pupil size (mm)	6	6	3
Pupil reaction to 1% Pilocarpine	No reaction	No reaction	No reaction
Pupil reaction to 1% Tropicamide	No reaction	No reaction	Dilates from 3 to 5 mm
Iris crypts	Absent	Absent	Absent
Pupillary filiform projection	Present	Present	Present
Retinal arteriolar tortuosity	Present	Present	Present
Retinal arteriolar occlusion	Absent	Absent	Absent
Retina venular course	Normal	Normal	Normal

*MRI* magnetic resonance imaging, *MRA* magnetic resonance angiography, *N/A* not applicable, *OD* right eye, *OS* left eye, *PDA* patent ductus arteriosus

**Fig. 2:**
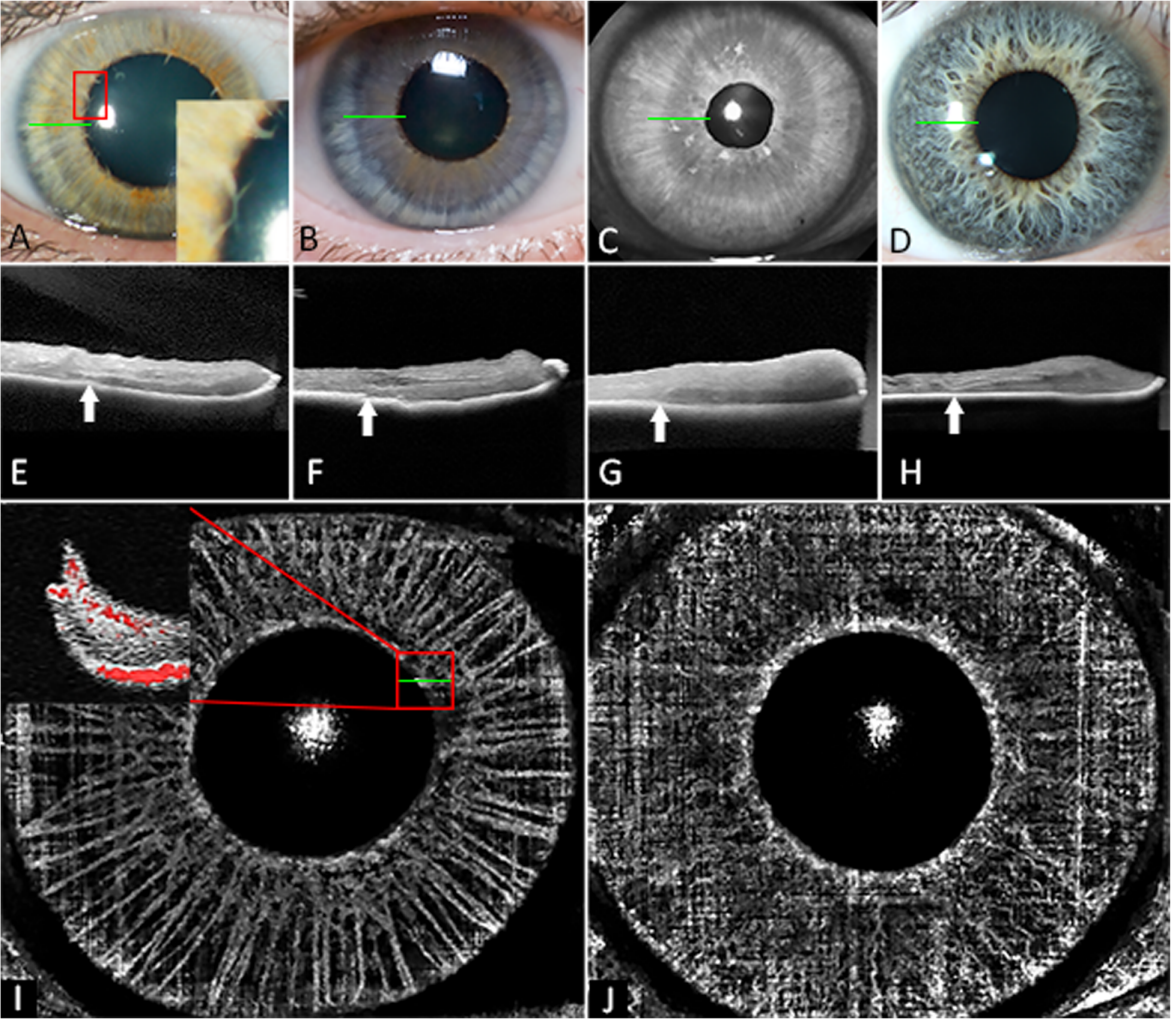
Colour photographs of the iris showing hypotrophy, loss of iris crypts, radial filiform projections (red box) and a central zone of iris discolouration in the proband (**a**) and her older sister (**b**). Infrared reflectance imaging of the proband’s mother’s iris showing partial pupillary constriction and a hyporeflective central zone in the iris suggesting the presence of a dysfunctional sphincter muscle (**c**). Normal iris has prominent crypts (**d**). Cross-sectional iris optical coherence tomography (OCT) shows hyporeflective layer anterior to the iris pigment epithelium in the central region of the iris in the proband (**e**), proband’s older sister (**f**), proband’s mother (**g**) and a control subject (**h**). En face iris OCT angiography of the proband’s older sister (**i**) showed paucity of iris vessels compared to a control subject (**j**) and the presence flow signal within a radial filiform projection (red box in **i**)

**Fig. 3:**
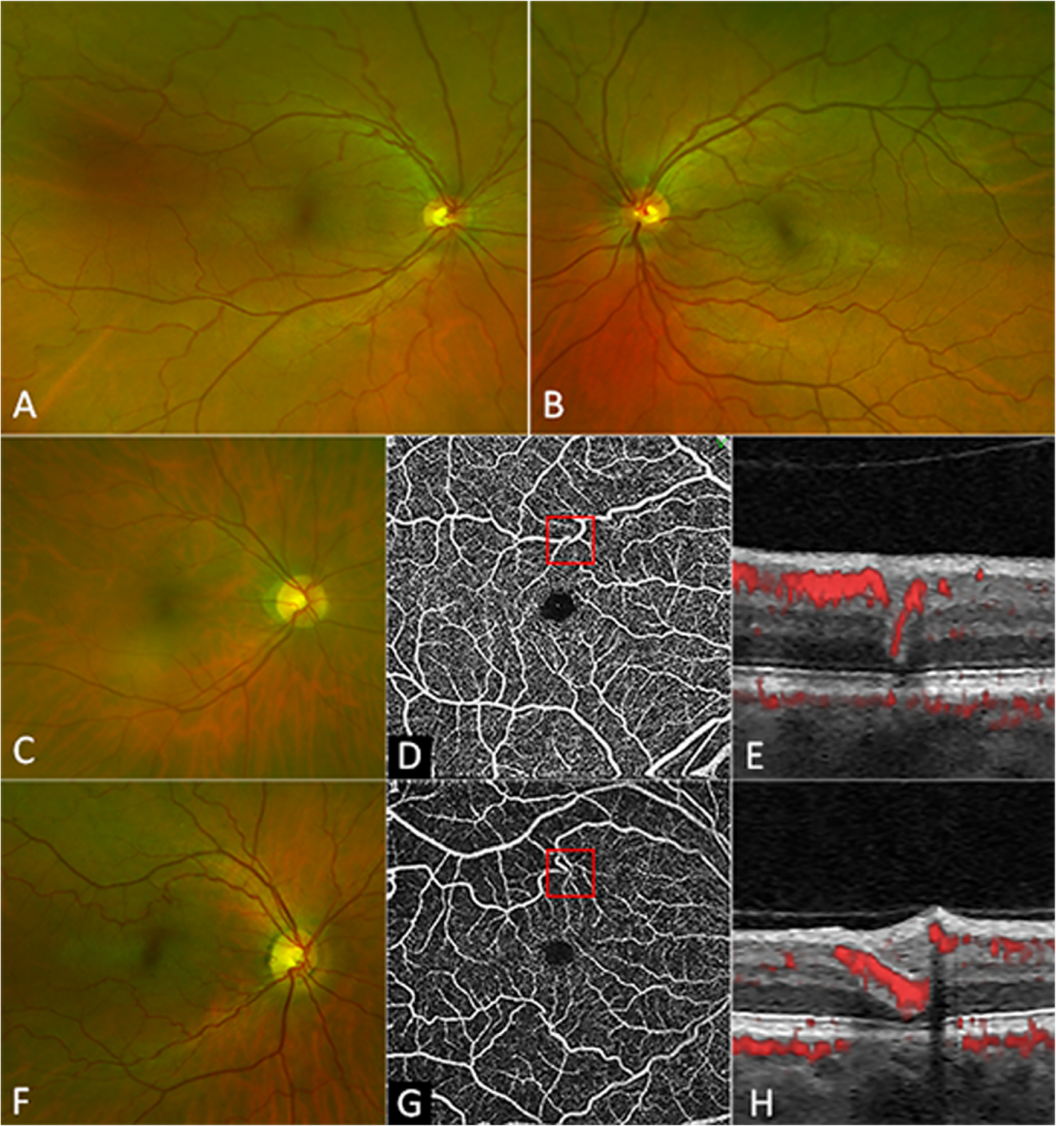
Widefield retinal photography showing corkscrew tortuosity of the retinal arterioles in the proband’s right (**a**) and left (**b**) eyes. The proband’s older sister has a similar retinal arteriolar tortuosity (**c**). En face optical coherence tomography angiography showing retinal arteriolar tortuosity (**d**) dipping into the outer nuclear layer without aneurysmal dilatation (**e**). The proband’s mother also has similar retinal arteriolar tortuosity (**f**, **g**) with the dipping of vessels into the outer nuclear layer (**h**)

Iris OCT showed absence of thickening in the central portion, a smooth anterior surface and a distinct hyporeflective layer just anterior to the iris pigment epithelium (IPE) near the pupillary border, coinciding with iris discoloration and hyporeflectivity on infrared reflectance imaging (Fig. 2). Iris OCTA (supplementary material 1) showed flow signal within the radial filiform projections and paucity and straightening of iris stromal vessels (Fig. 2). AO imaging (supplementary material 2) showed a similar mean (SD, range) age-adjusted wall-to-lumen ratio (WLR) of 0.27 (0.03, 0.23–0.29) and 0.24 (0.03, 0.20–0.29) between patients and controls suggesting lack of eutrophic remodelling (*p* = 0.24, supplementary material 3). Mean (SD, range) age-adjusted wall cross-sectional areas (WCSA) were 4.5 (1.4, 3.2–6.0) × 10^3^ μm^2^ in patients and 3.9 (1.2, 2.2–5.9) × 10^3^ μm^2^ in controls, but this difference was not statistically significant (*p* = 0.54) suggesting lack of hypertrophic remodelling in the immediate branches of the central retinal artery (supplementary material 3). Retinal OCTA showed no evidence of aneurysmal dilation but retinal arterioles were seen to dive into outer retinal layers as a result of excessive elongation of the vessel (Fig. 3).

In view of the dominant inheritance of the cerebral arteriopathy, the differential diagnosis included cerebral autosomal dominant arteriopathy with subcortical infarcts and leukoencephalopathy; CADASIL, related to *NOTCH3* mutations, and MMD-like cerebral arteriopathy in association with PDA and congenital mydriasis, related to *ACTA2* mutations. Although Sanger sequencing (Mutalyzer) of the hot spot regions for *NOTCH3* mutation (exons 2–8, 11, 18, 19, 22 and 23) in the proband (III:1) failed to show any pathogenic variants, a heterozygous sequence variant c.351C > G was identified in exon 4 of *ACTA2*. This variant results in an asparagine (AAC) to lysine (AAG) amino acid change at codon 117 (p.(Asn117Lys)). The same variant was identified in the heterozygous state in the affected sibling of the proband (III:3). It was not identified in the DNA sample from the father (II:4), but was present at a lower level, as judged by the height of the Sanger sequencing peak, in the DNA sample from the mother’s peripheral blood (II:5, supplementary material 4), indicating that the mother was a somatic mosaic. Although this sequence variant has not been described previously, it is considered likely to be pathogenic (ACMG Class 4): it is not present in gonomAD, it affects a highly conserved residue in a functional domain of the protein and in silico analysis software suggests it is disease-causing (VarSome). A family pedigree showing variant segregation, ocular and cerebral manifestation is summarized in supplementary material 5.

## Discussion and conclusions

We describe a novel mutation c.351C > G in *ACTA2* resulting in Asn117Lys substitution, associated with features of MSMDS including congenital mydriasis, PDA and cerebral arteriopathy in 2 sisters. Their mother had a milder ocular signs and asymptomatic cerebral arteriopathy. To the best of our knowledge, this is the first report that revealed novel iris and retinal features associated with MSMDS. Iris OCT indicated the presence of an iris sphincter muscle whilst iris OCTA confirmed flow signals within the filiform projections. There was no eutrophic or hypertrophic remodelling of the retinal arteriolar walls based on AO imaging and retinal OCTA confirmed elongation rather than aneurysmal dilatation of the retinal arterioles giving rise to the corkscrew appearance.

A key diagnostic test in the assessment of early-onset stroke associated with PDA and congenital mydriasis was MRI/MRA and catheter cerebral angiography as they demonstrated the features that differentiated *ACTA2*-related cerebral arteriopathy from MMD [13]. Classic MMD is characterized by bilateral occlusive disease of the terminal carotid or proximal middle cerebral arteries and basal collaterals contributing to the radiologic “puff of smoke” sign [20]. This is an important radiological finding that is associated with a high stroke recurrence risk [21]. However, it does not have the more widespread carotid involvement and proximal dilated vessel component seen in *ACTA2*-related cerebral arteriopathy. Munot et al. [13] described the typical cerebrovascular features of α-SMA p.(Arg179His) substitution as stenosis of the supraclinoid segment of both internal carotid arteries (ICAs) with dilatation of their proximal portions, straight cerebral vessels with focal stenosis and absence of basal collaterals. Regalado et al. expanded on the spectrum of Arg179 alterations that causes cerebral arteriopathy to include substitutions to cysteine, leucine and serine in addition to histidine [22]. Substitutions at two other residues, 46 and 258 have also been linked to this distinct form of cerebral arteriopathy. In contrast, previous reports of Asn117 substitution did not describe any cerebral vascular phenotype; p.(Asn117Thr) [11], p.(Asn117Ser) [23] and p.(Asn117Ile) [24] have been associated with familial or sporadic TAAD alone or uterine atony (supplementary material 6) [25]. However, each of these mutations have been associated with stroke at the age of 58 [11], 39 [23], and 44 years [24] without clinical details of cerebrovascular anatomy. Although most affected members had TAAD, none of these cases had documented iris abnormality (supplementary material 6). Another family with p.(Arg118Gln) had only 1 affected member manifesting cerebrovascular disease at the age of 57 [11, 26]. Interestingly, both p.(Asn117Thr) and p.(Asn117Ser) variants in the skeletal muscle alpha actin gene (*ACTA1*) have been associated with muscle disease multiple times [27] and in the Leiden Open Variant Database *ACTA1* Locus Specific database [28]. Therefore, this is the first report to demonstrate that, in addition to the commonly recognised substitutions at Met46 [29], Arg179 [13], and Arg258 [11], p.(Asn117Lys) can also cause *ACTA2*-related cerebral arteriopathy. It is intriguing that only one of the 3 affected members of this family developed stroke; the proband at the age of 11 and 30 years whilst her sister and mother had only MRI evidence of white matter ischaemia at the age of 41 and 66 years, respectively. The variation in cerebrovascular disease penetrance could be explained by differences in severity of cerebral arteriopathy and the degree of collateralization.

Fixed or non-reactive pupils is a universal finding in patients with MSMDS associated with ACTA2 Arg179 alterations [22]. Although clinically, this may be easily misdiagnosed as aniridia or partial aniridia at the bedside, careful slit lamp examination is sufficient to identify the key iris features of congenital mydriasis without in-depth imaging [19]. However, without high-resolution iris imaging, multiple reports have hypothesized that the congenital mydriasis in *ACTA2* mutation is due to absence of the sphincter muscle. The only published iris OCT in a case of congenital mydriasis showed iris thinning [18]. The new finding of a hyporeflective layer anterior to the IPE in the central zone suggesting sphincter muscle fibres are probably present but non-functional due to loss of contractility or fibrosis secondary to dysfunctional actin filaments, which is known to be expressed in these SMC. The ability to dilate the pupil in the mosaic mother (from 3 to 5 mm) further supports the presence of and activity in iris sphincter muscle. The filiform projections from the pupillary margins have been thought to be the remnants of the pupillary membrane. The presence of OCT flow signal within these projections suggests that they may represent incompletely regressed anterior tunica vasculosa of the hyaloid artery system. Fundal photography demonstrated significant retinal arteriolar tortuosity but no venular abnormality in all 3 patients. Retinal arteriolar tortuosity has been found in 35% of ACTA2 Arg179-related MSMDS and is a retinal feature associated with congenital mydriasis and not with iris flocculi, a separate iris phenotype of *ACTA2* mutation due to substitutions at Arg149 [22, 26]. Therefore, this is a useful sign for confirming MSMDS but not necessary for clinical diagnosis. The predilection of arteriolar rather than venular involvement may be related to the predominance of γ-SMA in the latter compensating for reduced polymerisation of α-SMA. Massive SMC proliferation in small, medium and large arterial walls has been illustrated in a histological study but it did not mention arteriolar wall changes [15]. The unique opportunity to examine arteriolar wall thickness in vivo allowed us to conclude that there was no evidence of the massive concentric SMC proliferation seen in histology of arterial walls despite elastin is also lacking in retinal arterioles. However, the corkscrew-like course into the outer nuclear layer of the neuroretina suggests that proliferation of SMC may occur in a co-axial direction leading to vessel elongation. Interestingly, a previous report documented the association between MMD and tortuous retinal artery but there was no detailed description of the iris, the cerebral vascular anatomy or genotyping to confirm if this case was related to *ACTA2* mutation [30].

The amino acid residue Asn117 is in the actin subdomain 1, and is therefore part of the barbed end of the G-actin monomer. This is the end of actin most often involved in elongation of actin filaments through actin polymerisation. Asn117 in particular is part of an alpha helix that interacts with cofilin, a regulator of filament dynamics which binds in between two adjacent actin subunits to promote disruption of the inter-monomer bonds. It could therefore be predicted that mutation of Asn117 might affect both actin polymerisation and depolymerisation [31]. Bergeron et al. modelled the effect of two human *ACTA2* mutants, p.(Asn117Thr) and p.(Arg118Gln) in yeast actin which is highly similar to smooth muscle actin [31]. Both mutations disrupted polymerisation. The p.(Arg118Gln) mutant sensitised actin filaments to cofilin, but the p.(Asn117Thr) actin mutant was resistant to the action of cofilin, suggesting a problem with both assembly and disassembly of actin filaments. It is not possible to be certain of what effect the p.(Asn117Lys) mutant might have on actin function, since, as Bergeron et al. [31] also discussed, different substitutions at the same amino acid residue in actin can have different functional consequences, depending on the precise molecular alterations introduced by the new amino acid. However, it is most likely p.(Asn117Lys) will affect polymerisation and/or depolymerisation. Analysis of the p.(Asn117Lys) mutant itself would be necessary to be sure of its impact on actin function.

In conclusion, this is the first report to demonstrate that p.(Asn117Lys) can cause a cerebral arteriopathy similar to those described in Met46, Arg179 and Arg258 substitutions. Iris imaging findings support the hypothesis that α-SMA Asn117Lys substitution lead to reduced SMC contractility in the iris sphincter muscle rather than its absence. Normal retinal arteriolar wall thickness suggests co-axial proliferation of smooth muscle cells in the retinal arteriolar wall resulting in vessel elongation and corkscrew tortuosity. Future phenotyping studies and histology are required to investigate whether other *ACTA2* mutations also lead to similar ocular features described herein.

## Supplementary information

**Additional file 1.** Supplementary Material 1: Optical coherence tomography angiography. Supplementary Material 2: Adaptive optics vessel wall thickness measurement. Supplementary Material 3: Scatter plot of wall-to-lumen ration and wall cross-sectional area. Supplementary Material 4: Sequencing chromatogram of control and 4 family members. Supplementary Material 5: Pedigree showing variant segregation, ocular and cerebral phenotype. Supplementary Material 6: A table showing various phenotypes associated with substitution at or near residue 117 of α-smooth muscle actin.

## Data Availability

All data generated or analysed during this study are included in this published article and its supplementary information files.

## Abbreviations

ACMG: American College of Medical Genetics and Genomics
ACTA2: Actin alpha 2
AO: Adaptive optics
APW: Aorto-pulmonary window
CT: Computed tomography
DNA: Deoxyribonucleic acid
ICA: Internal carotid artery
IPE: Iris pigment epithelium
MCA: Middle cerebral artery
MMD: Moyamoya disease
MRA: Magnetic resonance angiography
MRI: Magnetic resonance imaging
MSMDS: Multisystemic smooth muscle dysfunction syndrome
OCT: Optical coherence tomography
OCTA: Optical coherence tomography angiography
OMIM: Online Mendelian inheritance in man
PDA: Patent ductus arteriosus
SMC: Smooth muscle cell
TAAD: Thoracic aortic aneurysm and dissection
WCSA: Wall cross-section area
WLR: Wall-to-lumen ratio
α-SMA: Alpha-smooth muscle actin
γ-SMA: Gamma-smooth muscle actin
