# Supplementary material for: High-resolution iris and retinal imaging in multisystemic smooth muscle dysfunction syndrome due to a novel Asn117Lys substitution in ACTA2: a case report
Source: BMC Ophthalmol. 2020 Feb 24;20:68. doi: 10.1186/s12886-020-01344-w (PMC7038593; doi:10.1186/s12886-020-01344-w)
Supplement: Supplementary file 1 — Additional file 1. Supplementary Material 1: Optical coherence tomography angiography. Supplementary Material 2: Adaptive optics vessel wall thickness measurement. Supplementary Material 3: Scatter plot of wall-to-lumen ration and wall cross-sectional area. Supplementary Material 4: Sequencing chromatogram of control and 4 family members. Supplementary Material 5: Pedigree showing variant segregation, ocular and cerebral phenotype. Supplementary Material 6: A table showing various phenotypes associated with substitution at or near residue 117 of α-smooth muscle actin. [file 12886_2020_1344_MOESM1_ESM.docx]

**Supplementary Material**

**High-resolution iris and retinal imaging in multisystem smooth muscle dysfunction syndrome due to a novel Asn117Lys substitution in ACTA2: a case report**

Material 1: Optical coherence tomography angiography

Material 2: Adaptive optics vessel wall thickness measurement

Material 3: Scatter plot of wall-to-lumen ration and wall cross-sectional area

Material 4: Sequencing chromatogram of control and 4 family members

Material 5: Pedigree showing ocular and cerebral phenotype

Material 6: Phenotypes associated with substitution at or near residue 117 of α-SMA

References for supplementary materials

**Supplementary Material 1: Optical coherence tomography angiography**

Optical coherence tomography angiography (OCTA) was performed using the AngioVue Avanti XR (Optovue Inc, Fremont, USA) in AngioRetina mode. ReVue software (Version 2017.1.0.155) was used for reducing motion artefacts and en face view reconstruction.

For retinal vessel imaging, volume scans were acquired at vertical and horizontal meridians in 3 × 3 mm (304 × 304 B-scans), 6 × 6 mm (400 × 400 B-scans) or 8 × 8 mm (304 × 304 B-scans) grids centred at fovea in 2 patients (II:5 and III:3). Superficial and deep vascular complexes were segmented according to Hassan et al.^1^

For iris vessel imaging, the posterior segment objective lens was removed and the Z-axis was aligned to the anterior segment (i.e. iris surface). The autofocus was disabled to enable manual refocus to the iris plane. Volume scans in horizontal and vertical axes were acquired in a 6 × 6 mm (400 × 400 B-scans) region centred at the visual axis. Within the ReVue software, the slab boundaries were set anterior to the iris surface and posterior to the iris pigment epithelium, thus incorporating the entire iris tissue in all B-scans.^2,3^


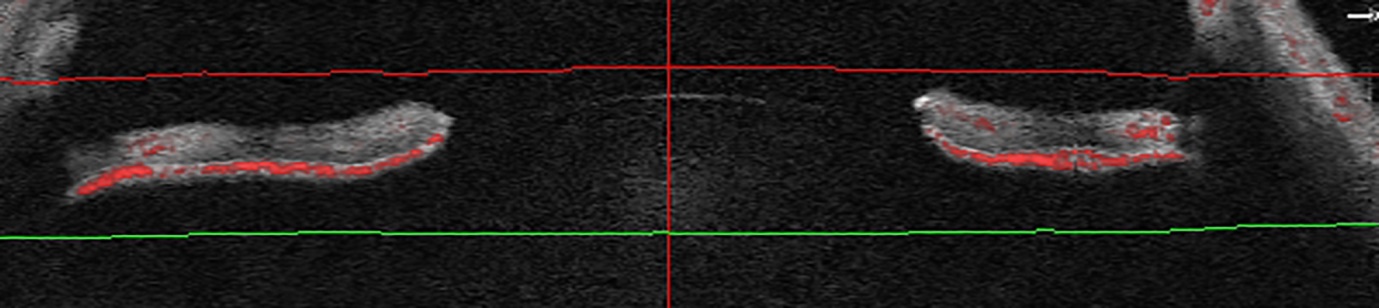


The anterior boundary is set in front of the iris surface whilst the posterior boundary is set behind the iris pigment epithelium (figure above). This ensures that signals from within the entire iris tissue are captured by the OCTA slab.

**Supplementary Material 2: Adaptive optics vessel wall thickness measurement**

Adaptive optics imaging of the retinal arteries was performed using a commercially available flood-illuminated adaptive optics camera (rtx1, Imagine Eyes, Orsay, France) in all patients (n=3) and 7 healthy age-matched controls. Overlapping 4° × 4° retinal images were taken at various locations surrounding and over the optic disc at a depth of 250 µm above retinal pigment epithelium.


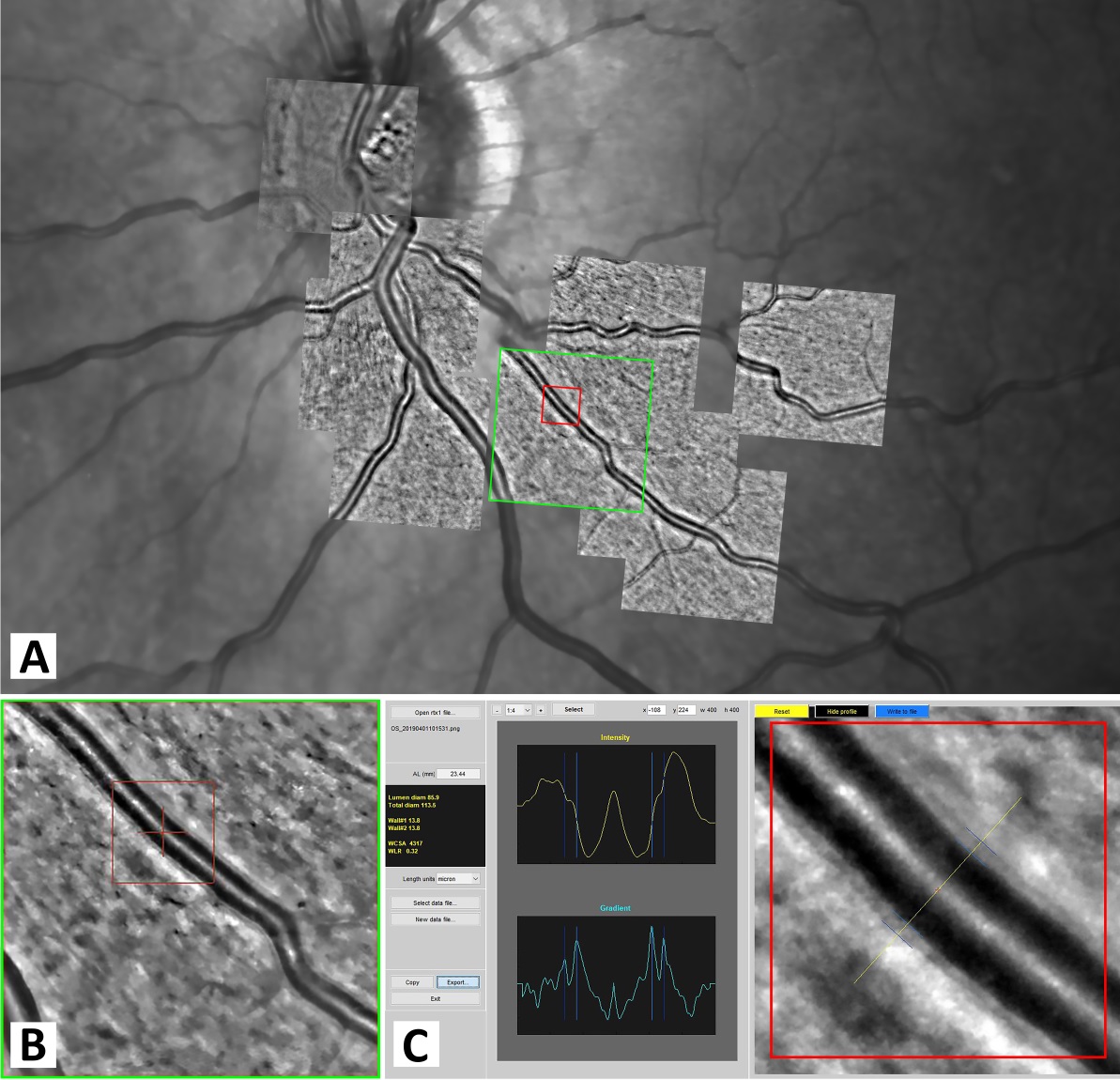


The figure above shows (A) montage of the adaptive optics images overlaid on near-infrared reflectance fundus image. Part (B) illustrates a retinal arteriole near optic disc is chosen and part (C) shows how the inner and outer vessel wall boundaries are marked by the AODetect software with manual adjustment if necessary.

**Supplementary Material 3: Scatter plot of wall-to-lumen ration and wall cross-sectional area**

Retinal arteriolar wall-to-lumen ratio (WLR) and wall cross-sectional area (WCSA) of the retinal arteries were measured using AODetect software (Imagine Eyes, Orsay, France) at locations approximately 1-2 disc diameter away from the optic disc margin. For each of the 3 subjects, 5 AO images were selected and 2 regions of interests from each image were used for measuring vessel diameters and wall thicknesses; i.e. 10 measurements per subject. Vessels wall was detected automatically by the software and adjusted manually if required. For the 7 healthy controls, 1-2 images were selected and 2 regions of interest from each image were used for measuring vessel diameter and wall thicknesses; a total of 24 measurements across 7 subjects. The WLR and WCSA were adjusted to the age of the patient by the method described by Meixner and Michelson.^4^ An independent sample t-test was used to compare the mean age-adjusted WLR and WCSA between patient and controls.


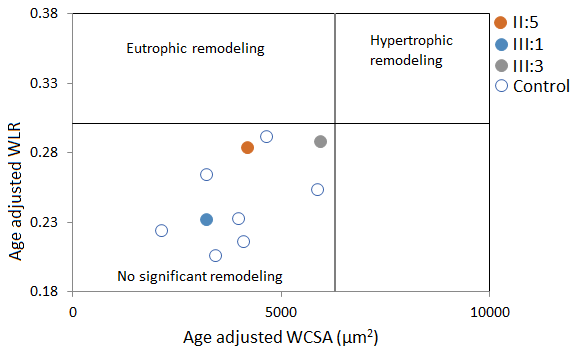


The scatter plot (figure above) shows the age-adjusted WLR and WCSA in the three patients and seven healthy controls. The reference lines indicate the mean plus 2×standard deviation of the healthy control values as reported by Meixner and Michelson.^1^ No eutrophic or hypertrophic remodelling was detected in patients/controls.

**Supplementary Material 4: Sequencing chromatogram of control and 4 family members**

The coding exons of the candidate gene smooth muscle aortic alpha-actin gene (ACTA2, OMIM #102620, accession number NM_001141945.1) were sequenced from genomic DNA extracted from peripheral blood, by Sanger sequencing (primer sequences available on request). Numbering of the amino-acid residues in ACTA2 was from the initiating methionine in accordance with Human Genome Variation Society recommendations. The pathogenicity of the identified amino acid substitution was assessed on the basis of the evolutionary conservation of the affected amino acid residue, minor allele frequency in healthy populations and by in silico prediction software (VarSome (<https://varsome.com>)) in accordance with the recommendations of the American College of Medical Genetics and Genomics.^5^


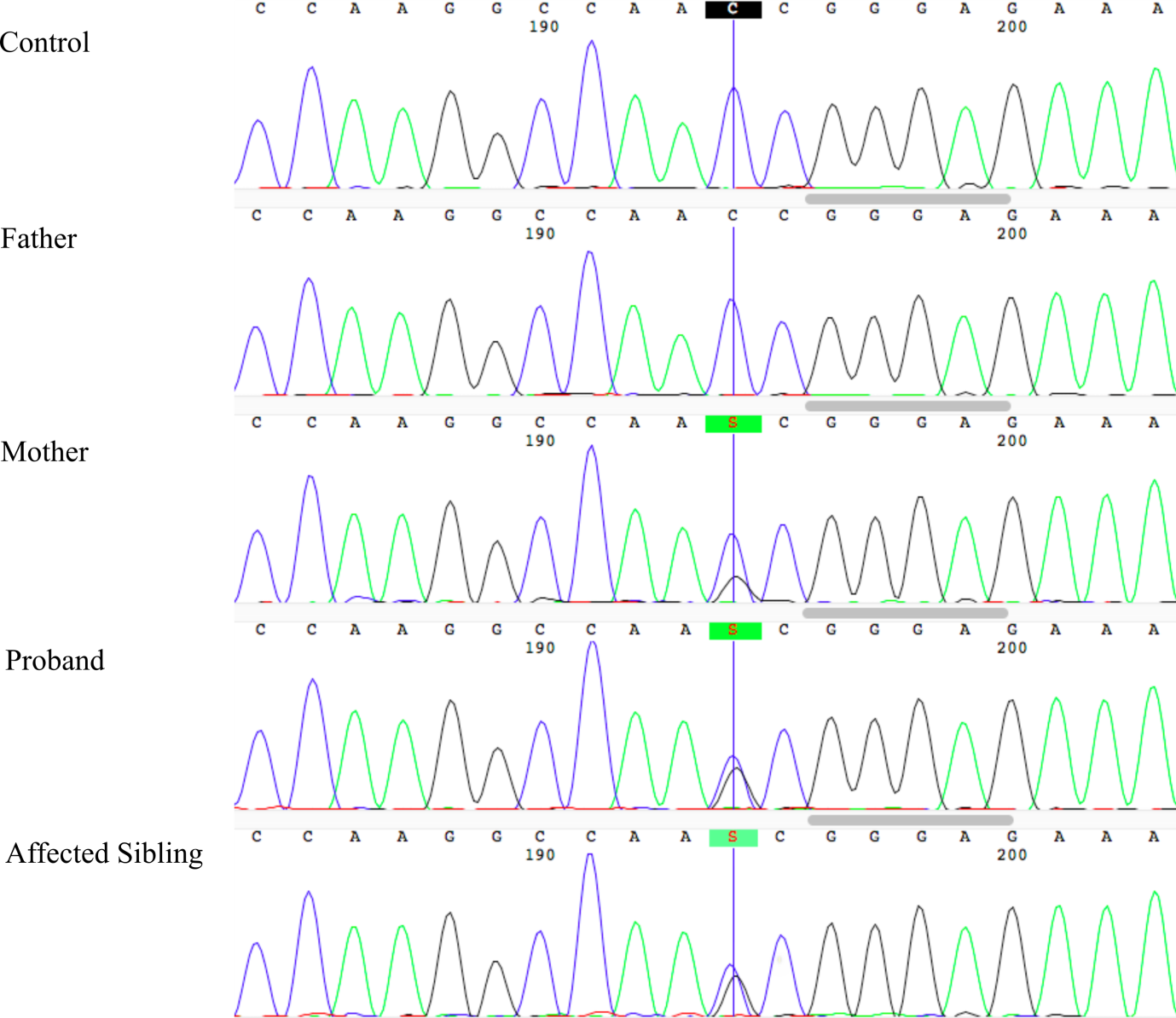


Sequencing chromatogram (above figure) shows the normal sequence and c.351C>G transversion in proband and her older sister, mosaic in the mother and no transversion in the father

**Supplementary Material 5: Pedigree showing variant segregation, ocular and cerebral phenotype**


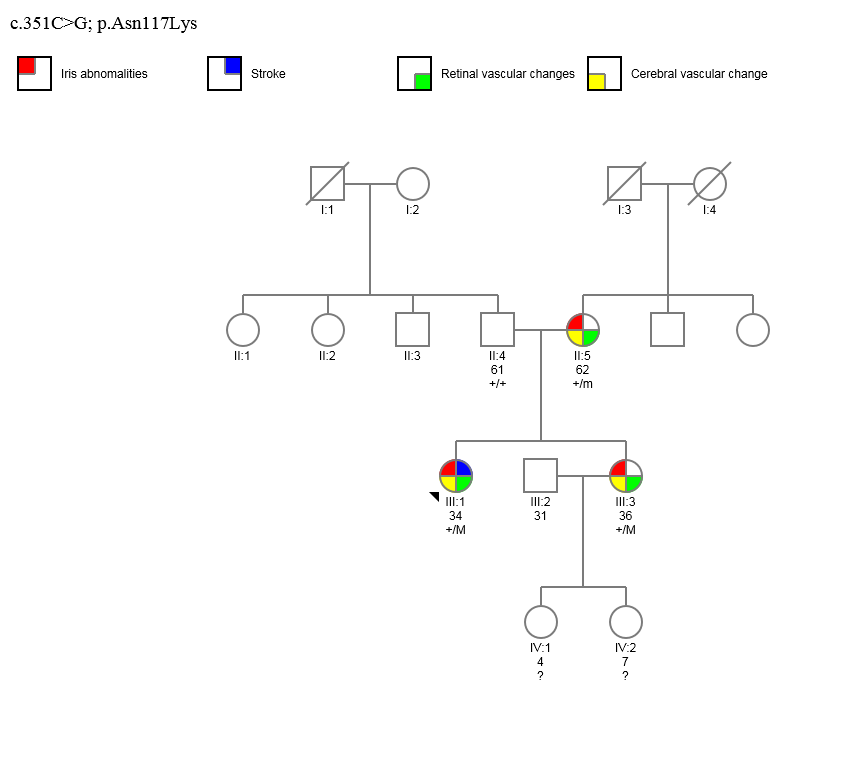
Pedigree showing all 3 family members manifesting iris, retinal and cerebral vascular anomaly but only the proband had symptomatic cerebral ischaemic event. Not shown is that only proband and her sister had patent ductus arteriosus repair.

**Supplementary Material 6: A table showing various phenotypes associated with substitution at or near residue 117 of α-smooth muscle actin**

| Author  (year) | Pedigree | Amino acid substitution/  Nucleotide change | Members (age of systemic phenotype onset) | Systemic features | Ocular features |
| --- | --- | --- | --- | --- | --- |
| Guo et al. (2007)^6^ | TAA039 | p.Asn117Thr/  c.397A>C | II:2 (71)  II:10 (56)  III:6 (36) | TAAD  TAAD  TAAD | None  None  None |
|  | TAA174 | p.Arg118Gln/  c.400G>A | I:2 (79)  II:4 (53)  II:8 (47) | TAAD  TAAD  TAAD | None  None  None |
| Guo et al. (2009)^7^ | TAA039 | p.Asn117Thr/  c.397A>C | II:2 (53)  II:4 (58)  II:9 (56)  III:6 (29) | TAAD  UC-CVD  TAAD  TAAD | None  None  None  None |
|  | TAA174 | p.Arg118Gln/  c.400G>A | II:2 (55/57)  II:3 (49/79)  III:2 (49)  III:3 (24) | TAAD/UC-CVD  CAD/deceased  TAAD  TAAD | None  None  None  None |
|  | TAA441 | p.Arg118Gln/  Not reported | I:2 (40/?)  II:2 (52/52)  II:3 (54/54)  III:6 (50/54/59)  III:8 (48)  III:10 (50)  III:12 (42/42)  IV:3 (28/28/28)  IV:8 (12/12) | CAD/deceased  CAD/deceased  CAD/deceased  TAAD/CAD/deceased  TAAD  CAD  TAAD/deceased  TAAD/CAD/deceased  TAAD/deceased | None  None  None  None  None  None  None  None  None |
| Poninska et al. (2016)^8^ | TAAD076 | p.Asn117Ser/  Not reported | II:2 (NA)  II:3 (39/42)  III:1 (20) | No details  UC-CVD/CAD  TAAD | None  None  None |
| Ke et al. (2016)^9^ | Family 1 | p.Asn117Ile/  c.825A>T | I:1 (44)  II:1 (39) | TAAD/UC-CVD  TAAD/AAAD | None  None |
| Cooper et al (2017)^10^ | Family 1 | p.Asn117Ser/  Not reported | Grandfather (40)  Father (19/21)  Daughter (26) | CAD  IAAD/TAAD  UA | None  None  None |
| **This report (2019)** | Family 1 | p.Asn117Lys/  c.351C>G | II:5 (62)  III:1 (0/32)  III:3 (0/34) | AR-CVD  PDA/AR-CVD  PDA/AR-CVD | CM/RAT  CM/RAT  CM/RAT |

AAAD, abdominal artery aneurysm and dissection; AR-CVD, *ACTA2*-related cerebrovascular disease ± stroke; CAD, coronary artery disease or “heart attack”; CM, congenital mydriasis; IAAD, iliac artery aneurysm and dissection; PDA, patent ductus arteriosus; RAT, retinal arteriolar tortuosity; TAAD, thoracic aortic aneurysm and dissection; UA, uterine atony; UC-CVD, uncharacterised cerebral vascular disease causing stroke;

**References**

1. Hassan M, Sadiq MA, Halim MS, Afridi R, Soliman MK, Sarwar S, et al. Evaluation of macular and peripapillary vessel flow density in eyes with no known pathology using optical coherence tomography angiography. Int J Retina Vitreous. 2017;3:27.

2. Allegrini D, Montesano G, Pece A. Optical Coherence Tomography Angiography in a Normal Iris. Ophthalmic Surg Lasers Imaging Retina. 2016;47:1138-9.

3. Roberts PK, Goldstein DA, Fawzi AA. Anterior Segment Optical Coherence Tomography Angiography for Identification of Iris Vasculature and Staging of Iris Neovascularization: A Pilot Study. Curr Eye Res. 2017;42:1136-42.

4. Meixner E, Michelson G. Measurement of retinal wall-to-lumen ratio by adaptive optics retinal camera: a clinical research. Graefes Arch Clin Exp Ophthalmol. 2015;253:1985-95.

5. Richards S, Aziz N, Bale S, Bick D, Das S, Gastier-Foster J, et al. Standards and guidelines for the interpretation of sequence variants: a joint consensus recommendation of the American College of Medical Genetics and Genomics and the Association for Molecular Pathology. Genet Med. 2015;17:405-24.

6. Guo DC, Pannu H, Tran-Fadulu V, Papke CL, Yu RK, Avidan N, et al. Mutations in smooth muscle alpha-actin (ACTA2) lead to thoracic aortic aneurysms and dissections. Nat Genet. 2007;39:1488-93.

7. Guo DC, Papke CL, Tran-Fadulu V, Regalado ES, Avidan N, Johnson RJ, et al. Mutations in smooth muscle alpha-actin (ACTA2) cause coronary artery disease, stroke, and Moyamoya disease, along with thoracic aortic disease. Am J Hum Genet. 2009;84:617-27.

8. Poninska JK, Bilinska ZT, Franaszczyk M, Michalak E, Rydzanicz M, Szpakowski E, et al. Next-generation sequencing for diagnosis of thoracic aortic aneurysms and dissections: diagnostic yield, novel mutations and genotype phenotype correlations. J Transl Med. 2016;14:115.

9. Ke T, Han M, Zhao M, Wang QK, Zhang H, Zhao Y, et al. Alpha-actin-2 mutations in Chinese patients with a non-syndromatic thoracic aortic aneurysm. BMC Med Genet. 2016;17:45.

10. Cooper K, Brown S. ACTA2 mutation and postpartum hemorrhage: a case report. BMC Med Genet. 2017;18:143.
